# Clinically Suspicious Cases of Hansen's Disease at a Tertiary Care Hospital in South India: A Clinicopathological Study

**DOI:** 10.7759/cureus.70661

**Published:** 2024-10-01

**Authors:** Asfia Shabnam, Leena Dennis Joseph, N Priyathersini, Anuradha Priyadarshini

**Affiliations:** 1 Department of Pathology and Laboratory Medicine, Sri Ramachandra Institute of Higher Education and Research, Chennai, IND; 2 Department of General Pathology, Sri Ramachandra University, Chennai, IND; 3 Department of Pathology and Laboratory Medicine, Sri Ramachandra Medical College and Research Institute, Chennai, IND; 4 Department of Dermatology, Sri Ramachandra Institute of Higher Education and Research, Chennai, IND

**Keywords:** clinical-histopathological concordance, hansen's disease, leprosy, mycobacterium leprae, wade-fite stain

## Abstract

Background and objective

Hansen's disease, also known as leprosy, is a chronic granulomatous condition caused by *Mycobacterium leprae*, primarily affecting the skin and peripheral nerves. The disease has a wide spectrum of clinical and histopathological manifestations, often mimicking other inflammatory and infectious conditions. This variability poses significant challenges in its early diagnosis and management. Aligning clinical suspicion with histopathological evidence is critical for effective treatment and control of transmission. This study was designed to explore the concordance between clinical impressions and histopathological diagnoses and to understand the diagnostic utility of various histopathological techniques. We aimed to establish and correlate the histopathological types of leprosy with clinical presentations. We also sought to determine the extent to which different histopathological techniques, including special stains, can corroborate with clinical types, thereby enhancing diagnostic precision.

Materials and methods

This was a hospital-based, retrospective study of clinically suspicious cases of leprosy at a tertiary care hospital in South India. A total of 100 cases of various age groups were identified and included in the study. All cases underwent skin biopsy with samples subjected to routine hematoxylin and eosin (H&E) staining and acid-fast bacilli (AFB) staining for the detection of lepra bacilli.

Results

Among the 100 clinically suspicious cases of leprosy, 50 were histopathologically confirmed as leprosy and 50 were categorized as non-specific dermatitis. Among the 50 histologically confirmed cases of leprosy, the most common type was lepromatous leprosy (LL) (38%) followed by borderline tuberculoid (BT) (30%) leprosy. Modified AFB stain was positive in 21 cases and was instrumental in confirming the suspected cases of leprosy. The overall correlation between the clinical and histopathological diagnosis was significant, with the highest correlation noted in LL cases.

Conclusions

Our findings underscore the complexity of diagnosing leprosy due to its varied clinical and pathological presentations. Despite a high overall concordance rate, the discrepancies between clinical impressions and histopathological findings observed highlight the need for a multidimensional diagnostic approach. Incorporating a combination of clinical assessment, routine histology, and special staining can enhance diagnostic accuracy, leading to better patient management and outcomes.

## Introduction

Leprosy or Hansen's disease is a chronic infectious disease caused by *Mycobacterium leprae* affecting millions worldwide. It has a notable prevalence in India, accounting for nearly 30% of global cases [1]. The pathogen targets several organ systems but mainly affects the skin, peripheral nervous system, and bone, manifesting in a variety of clinical forms [2]. Leprosy shows variations in the clinical presentation and histopathological interpretation of skin biopsy depending on the immunological status of the patient. The clinical diagnosis of leprosy is confirmed when acid-fast bacilli (AFB) in slit-skin smears (SSS) are demonstrated by Ziehl-Neelsen (ZN) staining. Histopathological examination of skin biopsies, supplemented by the detection of *Mycobacterium leprae*, constitutes a cornerstone in the diagnostic algorithm of leprosy. Histology not only aids in accurate classification but also provides insights into the disease progression and response to treatment. 

Despite its widespread recognition dating back to ancient times, the diagnosis of leprosy often relies heavily on clinical examination, particularly in rural and resource-limited settings [3]. This approach, while pragmatic, is fraught with challenges given the disease's diverse presentations and its penchant for mimicking other conditions, necessitating more reliable diagnostic strategies [4,5]. In light of these complexities and the critical need for precise diagnosis, this study aimed to underscore the importance of comprehensive histopathological examination coupled with advanced staining techniques in the diagnosis of challenging cases of leprosy, particularly where clinical diagnosis is inconclusive.

The abstract of this article was previously published in USCAP Modern Pathology 2021.

## Materials and methods

Study design and setting

This was a retrospective analytical study conducted at our tertiary care hospital spanning a period of two years from January 2017 to January 2019. All patients with suspected clinical diagnoses of leprosy visiting the dermatology inpatient and outpatient departments were enrolled in the study. The hospital information system was used to retrieve retrospective data about the enrolled patient’s demographics, clinical traits, disease spectrum, and SSS results.

Data collection

The leprosy cases were segregated clinically based on the Ridley-Jopling scale [6] and were subjected to biopsies from active skin lesions. A total of 100 skin biopsies were received in the histopathology department. The staining involved hematoxylin and eosin (H&E) stain and special staining by ZN stain for the identification of leprae bacilli.

Statistical analysis

The data collected was entered into a Microsoft Excel sheet (Microsoft Corp., Redmond, WA) and analyzed using IBM SPSS Statistics version 26 IBM Corp., Armonk, NY). Data were statistically described in terms of frequencies and percentages and presented using frequency tables.

## Results

This study involved a comprehensive clinicopathological examination of 100 clinically suspected cases of leprosy over two years, aiming to establish a correlation between clinical impressions and histopathological findings with a variety of lesion characteristics in terms of number, anatomical sites, nature, and color. The demographic data revealed that out of 100 cases, 78 were male patients and 18 were female patients (male-to-female ratio: 3.5:1); the cohort's median age was 46.5 years.

Clinical features and histopathological observations

**Figure 1:**
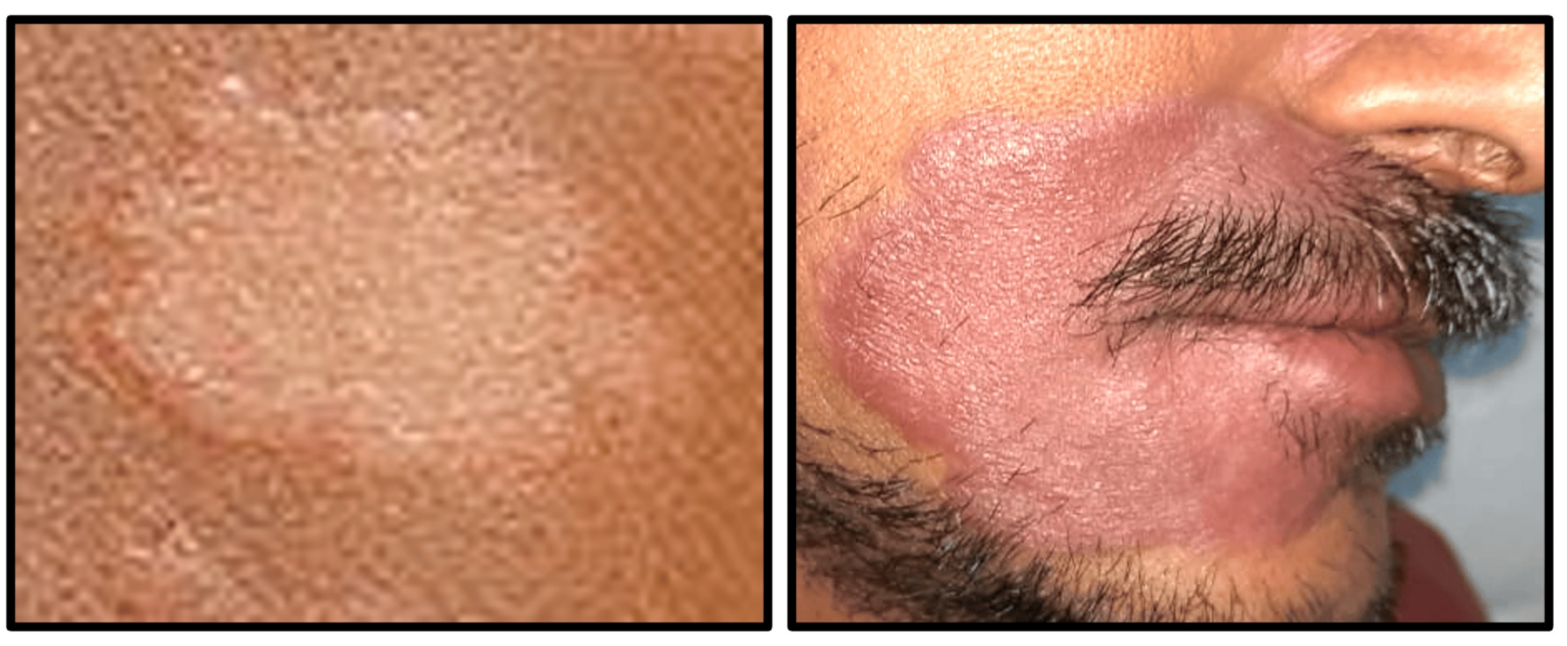
Clinical image showing a hypopigmented patch seen in a case of tuberculoid leprosy (left) and lepromatous leprosy (right)

Most patients presented with hypopigmented macular lesions (59 cases); plaques and papules were seen in 30 cases, nodules in 11 cases, and seven cases were deemed not clinically specified (Figure 1).

Of the 100 clinically suspicious cases of Hansen's disease, 50 were confirmed histopathologically as Hansen's disease, and 50 were categorized as non-specific dermatitis. The study utilized the Ridley-Jopling classification [6] for histopathological typing and applied modified acid-fast stains to detect *Mycobacterium leprae*. Clinically and histopathologically, most cases were in the lepromatous spectrum of leprosy. Concordance between clinical diagnosis and histopathology was found in 77% of cases, with the highest correlation seen in LL patients (92.85%). The most common subtype was LL (38%), followed by borderline tuberculoid (BT) (30%), tuberculoid (TT) (8%), and borderline lepromatous (BL) (6%) (Table 1).

**Table 1:** Distribution of clinicopathological factors among the cohort

Parameter		Number of cases
Sex	Female	22
	Male	78
Number of lesions	Single	18
	Multiple	75
	Unknown	7
Color of lesions	Hypopigmented	59
	Erythematous	41
Nature of lesions	Patch/macule	52
	Plaque/papules	30
	Nodules	11
	Unknown	7
Site	Upper limb	26
	Lower limb	17
	Upper limb and lower limb	6
	Face	12
	Chest and back	23
	Face, extremities, and trunk	14
Nerve involvement	Ulnar nerve	16
	Radial cutaneous nerve	4
	Great auricular nerve	4
	Common peroneal nerve	1
	Auriculotemporal nerve	1
	Median nerve	1
Loss of sensation	Decreased	45
	Intact	26
Hair loss	Present	10
	Absent	30

**Figure 2:**
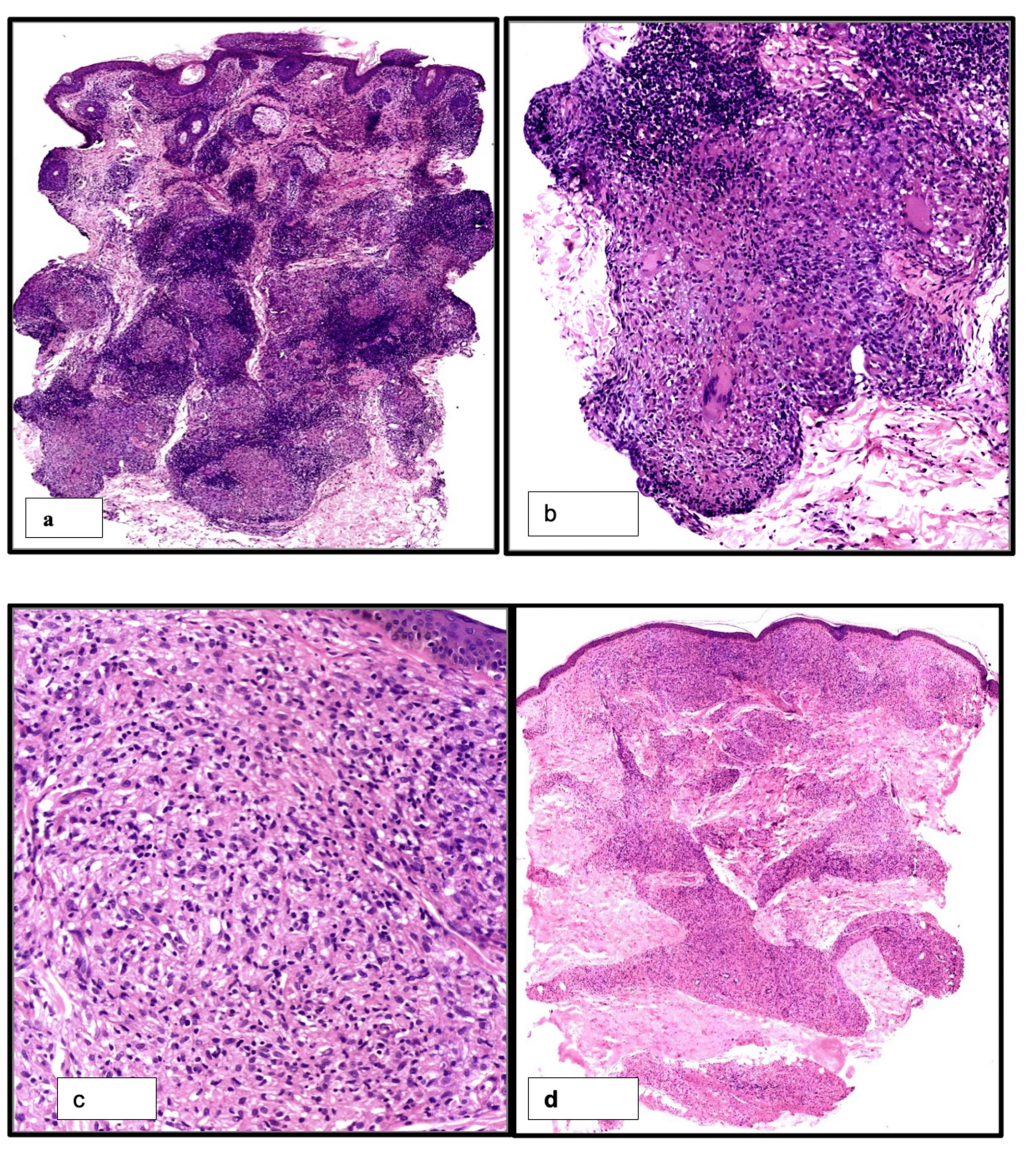
Tuberculoid leprosy with epithelioid histiocytes and granuloma formation (a, H&E,100x) with Langhans giant cell in the granuloma (b, H&E, 200x); microphotograph showing diffuse sheets of foamy macrophages (c, H&E 200x; d, 100x)

The majority of the cases belonged to the LL type while the BL type accounted for the least number of cases, indicating a spectrum of immune responses in the patient population (Figures 2a-2d). Nerve sensation was found to be decreased/lost in 45 cases, intact in 26 cases, and clinically not known in 29 cases. Nerve involvement was seen in 18 cases and the remaining 82 cases showed no nerve involvement. The most common nerve involved was the ulnar nerve (16%) followed by the radial nerve (4%), common peroneal nerve (2%), greater auricular nerve (1%), auriculotemporal nerve (1%), and median nerve (1%). Loss of hair was seen in 10 cases, absent in 39 cases, and clinically not known in the remaining 51 cases.

The modified acid-fast stain showed positivity in 21 cases, demonstrating its utility in confirming the presence of bacilli, especially in LL cases. However, its sensitivity was lower in non-lepromatous forms.

Dermatoses Mimicking Leprosy

A significant number of cases (51) were discordant in terms of correlation, highlighting the challenges in clinical diagnosis due to close clinical mimicking with other dermatoses. The non-specific inflammatory infiltrate and perivascular dermatitis were common patterns observed in discordant cases (Table 2).

**Table 2:** Histopathological diagnosis in clinically suspected lesions BL: borderline lepromatous; BT: borderline tuberculoid; IL: indeterminate; LL: lepromatous leprosy; PMLE: polymorphous light eruptions; TT: tuberculoid; PR: pityriasis rosea

Findings	Number of cases
LL	19
BL	3
TT	4
BT	15
IL	7
Non-specific dermatitis	35
Perivascular dermatitis	6
PMLE	3
Dermatitis	4
PR	1
Mastocytosis	1
Lupus	1
Total	100

## Discussion

Leprosy is a curable infectious disease caused by *Mycobacterium leprae*, and it continues to be endemic in >140 countries around the world. Although it was declared "eliminated" as a global public health problem by the World Health Organization in 2000, approximately 200,000 new cases were reported worldwide in 2017 [7]. The risk factors for leprosy include exposure through close contact with a recently diagnosed individual, exposure to armadillos, immunosuppression, and immunodeficiency.

In our study, out of 100 clinically suspicious cases of Hansen’s disease, 50 were confirmed histologically as leprosy and the remaining 50 were categorized as non-specific dermatitis. The demographic data revealed that 78 cases were male and 22 were female (male-to-female ratio: 3.5:1), with a median age of 46.5 years. Leprosy is believed to be more common in males as observed by Gupte et al. and Sehgal et al. [8,9]. Our results align with those of a study by Manandhar et al. [10], where 75% of patients were males. It may be due to urbanization and more opportunities for contact in males. The lower number of female patients may be due to the local social customs and taboos. Our findings contrast with those noted by Suri et al. [10], who reported a slightly higher female predominance with a male-to-female ratio of 1:1.1.

The most common skin lesions in the present study were hypopigmented macules followed by papules and plaques. This finding correlates with that of Manandhar et al. [10]. The ulnar nerve was the most common clinically thickened peripheral nerve (55%) in the present study. The Ridley-Jopling classification was used in our study to classify the cases. This classification categorizes leprosy into five types. In this study, clinically and histopathologically, most cases were in the lepromatous spectrum of leprosy. The most common clinical subtype was LL (n=19, 38%), followed by BT (n=15, 30%), tuberculoid (TT) (n=4, 8%), and BL (n=3, 6%). This pattern contrasts with the findings of Karre et al. [11] and Mehta et al. [12], where BT cases were the most common (29%) while mid-borderline cases were the least common (6%). We found a good correlation between clinical and histopathological findings in LL-type leprosy for 70.8% of cases. Our study noted nine cases (16%) of indeterminate leprosy. Our incidence rate was higher than those in previous studies, which could be attributed to the increased awareness of leprosy among people.

In our study, the Fite Faraco stain for AFB was found to be positive in 21 cases (52%) and negative in 27 (48%) cases. However, the stain was positive for all the cases of LL and BL cases. We observed a clinicopathological correlation in 50 skin biopsies (50%). Thus, the correlation between clinical and histopathological findings is considered pertinent for the precise typing of leprosy. Our study also highlights the challenges in clinical diagnosis due to close clinical mimicking with other dermatoses. A significant number of cases (50) were discordant. The non-specific inflammatory infiltrate (70%) and perivascular dermatitis (18%) were common patterns observed in discordant cases, followed by polymorphous light eruptions (3%), mastocytosis (2%), and lupus (2%).

Challenges and limitations

While the diagnosis of leprosy based on clinical findings relies on the gross appearance of lesions, such methods lack the precision and depth afforded by histopathological assessments. Eliciting a thorough history, including that relating to travel to or residence in a country where leprosy is endemic is crucial when considering a diagnosis of leprosy. We sought to look into the correlation between clinical assessments and histopathological analyses in diagnosing leprosy. Despite advancements in diagnostic technologies, the diverse clinical manifestations of the disease pose significant challenges in terms of accurate classification and treatment. This necessitates a newer, more comprehensive strategy that integrates clinical evaluation, histopathology, and advanced staining methodologies to overcome the challenge. However, discrepancies between clinical impressions and histopathological findings in cases of IL and BL leprosy highlight the complexity of diagnosis and the limitations of relying solely on clinical assessment.

This study highlights the pivotal role of amalgamating clinical and histopathological evaluations in diagnosing Leprosy. A heterogeneous approach integrating advanced staining methods and immunofluorescence studies enhances diagnostic precision, facilitating timely treatment initiation and preventing disease transmission. Continuous research endeavors aimed at identifying diagnostic algorithms and exploring innovative diagnostic methodologies are crucial for the reduction of the burden of leprosy. These findings have significant implications for clinical practice and improved patient outcomes.

Future prospects

Molecular diagnosis has significantly improved leprosy detection, offering greater sensitivity and specificity. The anticipated advancements include developing point-of-care tests, integrating molecular diagnostics into control programs, genotyping *Mycobacterium leprae*, identifying molecular markers for predicting disease progression, and utilizing artificial intelligence. These advancements hold the potential to revolutionize the fight against leprosy, leading to earlier diagnosis, more effective treatment, and ultimately, a leprosy-free world.

## Conclusions

Our findings emphasize the complexity and variability in clinical presentation and histological diagnosis of leprosy. There may be certain overlaps between different types of leprosy both clinically and morphologically. Hence, determining the correlation between clinical and histopathological features appears to be more useful for accurate typing of leprosy. Despite a notable concordance between clinical and histopathological diagnoses in our study, the presence of discordant cases underscores the necessity of employing a comprehensive diagnostic approach. We strongly advocate for the continuous collaboration between dermatologists and pathologists to ensure precise and early diagnosis, ultimately leading to better patient management and outcomes.
